# Expression of CD44 is associated with aggressiveness in seminomas

**DOI:** 10.1007/s11033-024-09638-8

**Published:** 2024-05-25

**Authors:** Vasiliki T. Labropoulou, Dimitra Manou, Panagiota Ravazoula, Fatimah Mohammed Alzahrani, Haralabos P. Kalofonos, Achilleas D. Theocharis

**Affiliations:** 1 Department of Internal Medicine, Division of Hematology, University of Patras Medical School, Patras, Greece; 2 Biochemistry, Biochemical Analysis and Matrix Pathobiology Research Group, Laboratory of Biochemistry, Department of Chemistry, University of Patras, Patras, Greece; 3 Department of Pathology, University Hospital of Patras, Patras, 26504 Greece; 4 Department of Chemistry, College of Science, Princess Nourah bint Abdulrahman University, P.O. Box 84428, Riyadh, 11671 Saudi Arabia; 5 Clinical Oncology Laboratory, Division of Oncology, Department of Medicine, University of Patras, Rio, 26504 Greece

**Keywords:** Testicular germ cell tumors, Seminoma, CD44, Extracellular matrix, Tumor microenvironment, Hyaluronan receptor

## Abstract

**Background:**

Testicular germ cell tumors (TGCTs) exhibit diverse biological and pathological features and are divided in two main types, seminomas and nonseminomatous germ cell tumors (NSGCTs). CD44 is a cell surface receptor, which is highly expressed in malignancies and is implicated in tumorigenesis affecting cell-matrix interactions and cell signaling.

**Methods and results:**

Here, we examined the expression of CD44 in tumor cell lines and in patients’ material. We found that CD44 is over-expressed in TGCTs compared to normal tissues. Immunohistochemical staining in 71 tissue specimens demonstrated increased expression of CD44 in some patients, whereas CD44 was absent in normal tissue. In seminomas, a high percentage of tumor and stromal cells showed cytoplasmic and/or cell surface staining for CD44 as well as increased staining for CD44 in the tumor stroma was found in some cases. The increased expression of CD44 either in tumor cells or in stromal components was associated with tumor size, nodal metastasis, vascular/lymphatic invasion, and disease stage only in seminomas. The increased stromal expression of CD44 in TGCTs was positively associated with angiogenesis.

**Conclusions:**

CD44 may exhibit diverse biological functions in seminomas and NSGCTs. The expression of CD44 in tumor cells as well as in tumor stroma fosters an aggressive phenotype in seminomas and should be considered in disease treatment.

**Supplementary Information:**

The online version contains supplementary material available at 10.1007/s11033-024-09638-8.

## Introduction

Testicular germ cell tumors (TGCTs), although relatively rare are among the most common solid malignancies in young male adults and exhibit an increased incidence the last decades [1]. They are divided in two major histological types, homogeneous seminomas and heterogeneous non-seminomatous germ cell tumors (NSGCTs). Seminomas originate from undifferentiated germ cells and present more favorable outcomes compared to NSGCTs. NSGCTs comprise several subtypes such as embryonal carcinoma, teratoma, yolk sac tumor, choriocarcinoma and mixed type tumors consisting of two or more components and originate from undifferentiated and differentiated multipotent cells [2, 3]. Patients with TGCTs show overall a good response to platinum-based therapies with over 90% rate of cure in early diagnosed patients independent of histological type [4, 5]. Some patients are refractory to this treatment and present unfavorable clinical outcomes [6, 7].

TGCTs exhibit aneuploidies and few somatic mutations compared to other malignancies, which are involved in the development of the disease [8]. Additionally, the isochromosome 12p is considered as a marker of TGCTs and is present in almost all tumors. TGCTs present distinct epigenetic events compared to somatic cancers such as pluripotent-like DNA methylation patterns that also include hypomethylation of some histotypes [9]. DNA methylation is associated with acquired cisplatin resistance and therapeutic response to poly-ADP ribose polymerase (PARP) inhibitors as shown by the methylation status of several genes in TGCTs [9–11]. TGCTs exhibit a complex heterogeneity and is important to disclose the different oncogenic events implicated in the development and progression of the disease to optimize treatment and management. For example, the most frequent mutated oncogenes found in seminomas are *KIT* and *KRAs* whereas mutations in genes such as *PTMA*, *LZTR1* and *SRCAP* are present in NSGCTs [9].

In this context, we have investigated the role of extracellular matrix (ECM) molecules in TGCTs biology. ECM is a three-dimensional functional scaffold comprised by numerous interconnected macromolecules such as collagens, elastin, proteoglycans, hyaluronan, fibronectins and numerous (glyco)proteins. During tumorigenesis the crosstalk between tumor and stromal cells drives the remodeling of ECM creating a favorable microenvironment for tumor cell growth and spread [12–14]. Modified tumor ECM interacts through multiple receptors, including CD44, on the cell surface of both tumor and stromal cells permitting intracellular signals that regulate cell responses and phenotype [15–17]. Our previous studies have demonstrated that accumulation of ECM proteoglycan versican is associated with the metastatic potential of TGCTs [18], whereas stromal staining in seminomas and reduced levels of cell surface proteoglycan syndecan-4 in tumor cells in NSGCTs are indicators of increased aggressiveness [19]. Increased invasiveness of TGCTs cells is also associated with elevated expression of ECM remodeling enzymes matrix metalloproteinase-2 (MMP-2) and matrix metalloproteinase-9 (MMP-9) [20, 21]. Furthermore, membrane vesicles enriched in EMMPRIN/CD147 produced by aggressive TGCT cells such as embryonal carcinoma NT2/D1 cells regulate the expression and secretion of MMP-2 by fibroblasts to facilitate ECM remodeling and tumor spread [22].

Cell matrix interactions mediated by CD44 have been shown to activate tumorigenic signaling and drive tumor progression through diverse molecular mechanisms. CD44 is a transmembrane glycoprotein that binds to several ECM components including hyaluronan, fibronectin, laminin, proteoglycans such as serglycin and biglycan, growth factors and cytokines [12, 23, 24]. Its cytoplasmic tail is short and devoid of enzymatic activity, but it contains structural motifs involved in interactions with cytoskeletal and signaling proteins [12]. Additionally, it co-operates with several receptors including receptor tyrosine kinases as hepatocyte growth factor receptor (HGFR/MET), vascular endothelial growth factor receptor-2 (VEGFR-2), epidermal growth factor receptor (EGFR), G protein coupled receptors (GPCRs) and toll-like receptors (TLRs) interfering with multiple signaling pathways [12, 24]. Previous studies have shown that CD44 is expressed in seminomas and not in normal germ and stromal cells and in peritubular connective tissue [25, 26]. In another study with a limited number of patients, CD44 expression was also detected in NSGCTs and interestingly it was reported that apart from the standard isoform of CD44 (CD44s) expressed in seminomas, a high molecular weight isoform of CD44 (CD44v8-10) was expressed in NSGCTs [27]. Although CD44 is considered as a versatile tumor-promoting cell surface receptor its contribution to TGCTs biology has not been evaluated. We went on to evaluate the expression of CD44 in TGCTs cell lines and patients’ material and to examine the association of CD44 with the clinicopathological variables of the patients. We found that the elevated expression of CD44 in seminomas and stromal staining are related to tumor aggressiveness only in seminomas. Stromal CD44 staining also promotes neovascularization. All these data reveal diverse biological functions of CD44 in the progression of seminomas and NSGCTs.

## Materials and methods

### Cell lines and cultures

Mediastinal germ cell tumor embryonal carcinoma cell line NCCIT and NTERA-2/D1(NT2/D1) testicular embryonal carcinoma cell line were obtained from American Type Culture Collection (ATCC, Manassas, VA, USA). JKT-1 human seminoma cell line was a generous gift from Patrick Fenichel (University of Nice- Sophia-Antipolis, Faculty of Medicine, Nice, France). JKT-1 cells were cultured up to 38 passages to avoid the drift of these cells. Early passages of JKT-1 cells used in our study express a signature of seminoma markers (placenta alkaline phosphatase, NANOG, OCT3/4, AP2γ, and HIWI), which is still near the one expressed by seminoma cells, allowing their use as a model to study seminomas [28]. JKT-1 and NTERA-2/D1(NT2/D1) cell lines were cultured in DMEM, while NCCIT cell line was cultured in RPMI 1640 in a humidified atmosphere containing 5% CO2 at 37∘C. All culture media were supplemented with 10% fetal calf serum, 100 UI/mL penicillin and 100UI/mL streptomycin. Fetal calf serum was purchased from PAA Laboratories, Les Mureaux, France.

### Immunoblotting

Confluent cell cultures were lysed with lysis buffer containing 50 mM Tris pH 7.5, 150 mM NaCl, 5 mM EDTA, 1%Triton X-100 και 1% NP40, in the presence of a cocktail of protease inhibitors (Protease Inhibitor Cocktail Set V, Calbiochem). Equal amounts of proteins were reduced with β-mercaptoethanol in Laemmli buffer, separated by SDS-PAGE and transferred to polyvinylidene difluoride membranes (Macherey-Nagel). The membranes were blocked with 5% (w/v) non-fat dry milk in phosphate buffered saline (PBS) pH 7.4 containing 0.05% Tween-20 (PBS-T) and then probed with primary antibody for 20 h at 4 °C. Detection of the bound antibody was performed with peroxidase-conjugated secondary goat anti-mouse IgG (A4416, Sigma-Aldrich, Inc) for 90 min at room temperature and visualized by chemiluminescence (ECL, Amersham). Primary CD44 monoclonal antibody (Hermes-3, a kind gift from Prof. P. Heldin, Uppsala University) diluted 1:3000 in PBS-T containing 1% (w/v) non-fat milk powder.

### RNA isolation, cDNA synthesis and real-time qPCR analysis

Total RNA was isolated from cells using NucleoSpin® RNA kit (Macherey-Nagel) following the manufacturer’s instructions. The isolated RNA was quantified by measuring the absorbance at 260 nm. cDNA synthesis was performed using PrimeScriptTM RT Reagent kit (Perfect Real Time PCR) (TAKARA) according to manufacturer’s protocol. Real-Time qPCR analysis was conducted using the reaction mixture KAPA SYBR® Fast qPCR kit Master Mix (2x) Universal (KAPABIOSYSTEMS) according to manufacturer’s instructions using gene specific primers in a Rotor Gene Q equipment (Qiagen, USA). Relative quantification of the data was obtained using the ΔΔCt method and the GAPDH gene as normalizer. The Ct value of CD44 was normalized to the Ct of the normalizer (GAPDH). Fold changes (arbitrary units) were determined as 2-ΔΔCt. Primer sequences of the tested genes were 5’-CTTCAATGCTTCAGCTCCACCT-3’ and 5’-GACATAGCGGGTGCCATCAC-3’ for CD44 and 5’-AGGCTGTTGTCATACTTCTCAT-3’ and 5’-GGAGTCCACTGGCGTCTT-3’ for GAPDH.

### Patients and tissue samples

A retrospective study was performed including 71 patients with TGCTs (33 patients with seminomas and 38 with NSGCTs) who had undergone orchiectomy in our hospital. Patients were further treated according to the histological type, stage, and predictive and prognostic factors. Patients with stage I seminoma were treated with 2 cycles of adjuvant chemotherapy based on carboplatin. Patients with stage I NSGCTs were treated with 2–4 cycles of chemotherapy (bleomycin, etoposide, and carboplatin). Patients with stage II disease were treated with 4 cycles of adjuvant chemotherapy based on bleomycin, etoposide, and carboplatin, while in patients with stage III disease ifosfamide was added in the treatment pattern. Patients with NSGCTs with identified residual disease after completion of adjuvant chemotherapy underwent retroperitoneal lymph node dissection.

Tissue samples were obtained from the archive of the Pathology Department of the University Hospital of Patras. None of the patients had received prior chemotherapy or irradiation. The characteristics of the patients are summarized in Table 1. The staging of the tumour and the histopathologic findings were evaluated according to the American Joint Committee on Cancer. The study was performed in accordance with the principles established by the Helsinki Declaration. The study was approved by the Institutional review Board of the Medical School of the University of Patras and patients were enrolled after giving written consent. All data were analyzed anonymously.

**Table 1:** Clinicopathological variables of 71 patients with TGCTs

Variable	*n*	%
Histological type		
Seminoma	33	46.5
*Median age: 35 years*		
Non-seminoma	38	53.5
*Median age: 26 years*		
Embryonal carcinoma	8	11.3
Teratoma	5	7.0
Mixed type	25	35.2
Tumour size (T)		
T_1_	26	36.6
T_2_	41	57.7
T_3_	4	5.6
Vascular-lymphatic invasion		
Negative	32	45.1
Positive	39	54.9
Nodal status (N)		
N_0_	36	50.7
N_1_	9	12.7
N_2_	22	31.0
N_3_	4	5.6
Distant metastases (M)		
M_0_	63	88.7
M_1_	7	9.9
M_2_	1	1.4
Stage		
I	36	50.7
II	27	38.0
III	8	11.3

### Immunohistochemistry

Serial 5 𝜇m sections were taken from tissue samples embedded in paraffin and deparaffinized with xylene and dehydrated with 98% ethanol. Sections were incubated in 10mM citric acid buffer (pH 6.0) and heated in a microwave oven to perform antigen retrieval. The endogenous peroxidase activity was quenched with 3% hydrogen peroxide for 5 min at room temperature. Then, sections were blocked with 3% normal swine serum in PBS for 20 min at room temperature. Sections were incubated with a polyclonal antibody against CD44 (rabbit, H-300 Santa Cruz, USA) diluted 1:150 in PBS containing 1% normal swine serum for 1 h at room temperature. Then, sections were incubated with a biotinylated goat anti-rabbit antibody diluted 1:200 and the immune complexes were visualized by avidin-biotin-peroxidase technique (Dako Co., Copenhagen, Denmark). Staining was developed with 3,3-diaminobenzidine (DAB)/hydrogen peroxide for 5 min at room temperature and the sections were counterstained with hematoxylin. Positive tissue control and negative reagent control (without primary antibody) were run in parallel. The score of CD44 staining of tumor and stromal cells was estimated by measuring the percentage of CD44 positive cells into three groups: high staining > 30% of the cells stained; low staining 10–30% of the cells stained; and negative staining < 10% of the cells stained. CD44 staining in the tumor stroma was classified as follows: 0, no staining; 1+, moderate; 2+, strong staining. Overall, the levels of CD44 staining in stromal components was graded by scoring the percentage of CD44 positivity into two groups: negative (< 10% of stromal cells and negative staining of the stroma) and positive (> 10% of stromal cells or/and moderate or strong staining of the stroma).

The number of microvessels in each section was measured following staining of endothelial cells. Endothelial cells in tumour tissues were stained immunohistochemically with a rabbit polyclonal antibody against von Willebrand factor (A0082, Dako Co., Copenhagen, Denmark) as described by Imazono et al. [29]. The number of microvessels was measured in six random fields in each section and the mean value was estimated. The specimens were randomly evaluated by three independent researchers.

### Datasets

The results on the expression of CD44 in 136 patients with TGCTs (68 seminomas and 68 NSGCTs) and 165 normal tissues were obtained by GEPIA2 (Gene Expression Profiling Interactive Analysis 2, http://gepia2.cancer-pku.cn/#index, TGCT dataset, match TCGA normal and GTEx data). DNA methylation status of 149 patients with GCTs (63 seminomas and 86 NSGCTs) was obtained by cbioportal (https://www.cbioportal.org/, Human Methylation 27 (HM27) and Human Methylation 450 (HM450) merged arrays, Testicular Germ Cell Tumors, TCGA, PanCancer Atlas) [30].

### Statistical analysis

Data were analyzed using GraphPad Prism 5 (GraphPad Software). Statistically significant differences were evaluated using the two-tailed Fisher’s exact test to analyze the association between clinicopathologic variables and CD44 expression. Statistical significance was set at 𝑃 < 0.05. To evaluate the data of qRT-PCR analyses and the correlation of microvessel number with stromal expression of CD44, a two-tailed Student’s t-test was applied. Three independent biological samples have been used in each experimental set. Data are expressed as mean ± standard deviation (SD).

## Results

### CD44 is overexpressed in TGCTs

**Fig. 1:**
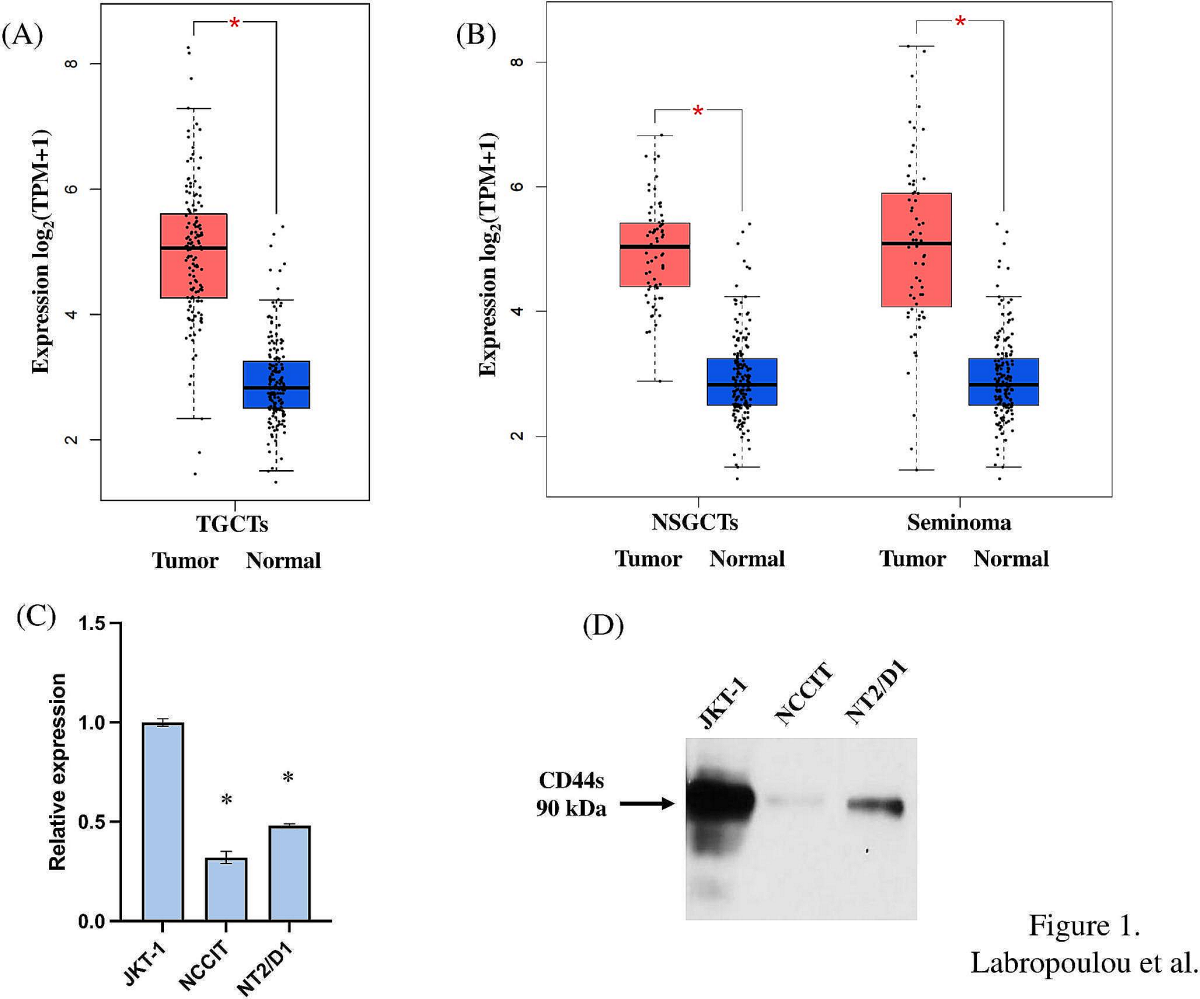
CD44 is highly expressed in TGCTs. (**A**) CD44 is overexpressed in TGCTs and distinct subtypes, seminomas and NSGCTs (**B**) compared to normal tissue. CD44 expression in 137 patients with TGCTs (68 seminomas and 68 NSGCTs) and 165 normal tissues were obtained by GEPIA2 (Gene Expression Profiling Interactive Analysis 2, http://gepia2.cancer-pku.cn/#index, TGCT dataset, match TCGA normal and GTEx data). (**C**) Relative mRNA levels of CD44 in JKT-1 seminoma, NCCIT and NTERA-2/D1(NT2/D1), embryonal carcinoma cells. The data are presented as the mean ± SD values (*n* = 3). Asterisk (*) indicates statistically significant differences (*p* ≤ 0.05) compared to JKT-1 cells. (**D**) Western blot analysis of protein levels of CD44 in JKT-1 seminoma, NCCIT and NTERA-2/D1(NT2/D1), embryonal carcinoma cells

We mined data on the expression of CD44 in 137 patients with TGCTs, including 68 seminomas and 68 NSGCTs and 165 normal tissues by using Gene Expression Profiling Interactive Analysis 2 (GEPIA2). Relative expression analysis of GEPIA2 online software, based on the TCGA RNA-seq datasets. According to the GEPIA2 data analysis, CD44 exhibits significantly higher expression in TGCTs samples compared to normal samples (Fig. 1A). The expression of CD44 is significantly higher either in seminomas or in NSGCTs samples compared to normal tissues (Fig. 1B). No significant differences in the expression of CD44 are present between seminomas and NSGCTs (Supplementary Figure S1A). We also analyzed the association of CD44 expression with the DNA methylation status of 63 seminomas and 86 NSGCTs by searching cbioportal (https://www.cbioportal.org/, Testicular Germ Cell Tumors, TCGA, PanCancer Atlas) [30]. By evaluating HM27 and HM450 arrays to measure the level of methylation at known CpG sites of CD44 gene we found an inverse correlation between methylation of the 5’UTR in the first exon and in the gene body and CD44 expression in seminomas. Interestingly, we noticed a positive correlation between methylation from 0 to 200 bases upstream of the transcriptional start site (TSS200) and CD44 expression in seminomas (Supplementary figure S1B). Similarly, we found an inverse correlation between methylation of the 5’UTR in the first exon and in the gene body and CD44 expression in NSGCTs (Supplementary figure S1C).

Then, we examined the mRNA and protein expression levels of CD44 in germ cell tumor cell lines. We found that all tumor cells expressed CD44 and the higher mRNA levels were detected in seminoma JKT-1 cells compared to embryonal carcinoma NTERA-2/D1(NT2/D1), and NCCIT cells (Fig. 1C). Western blot analysis confirmed that JKT-1 seminoma cells also expressed higher protein levels of the standard isoform of CD44 (CD44s) compared to NTERA-2/D1(NT2/D1) and NCCIT cells (Fig. 1D, Supplementary figure S1D). Higher molecular weight isoforms of CD44 were not detected in tumor cell lines, whereas lower molecular weight bands of CD44 were detected in JKT-1 cells that may represent fragments of the CD44s isoform.

### Overview of clinicopathological characteristics of patients with TGCTs

To evaluate the biological importance of the increased expression of CD44 in TGCTs, a retrospective study for the expression of CD44 in 71 patients was performed. The clinicopathological variables of the patients are summarized in (Table 1). Seminomas comprised 46.5% of the cases (33 patients with median age of 35 years at the time of surgery), while NSGCTs consisted of 53.5% of the cases (38 patients with median age of 26 years at the time of surgery). Patients with NSGCTs were classified into three groups: 8 (11.3%) with embryonal carcinoma, 5 (7.0%) with teratoma, and 25 (35.2%) with mixed type TGCTs. Twenty-six of the patients were of 𝑇1 stage, whereas 41 and 4 patients were of 𝑇2 and 𝑇3 stage, respectively. Thirty-nine patients were positive for vascular and/or lymphatic invasion. In 35 patients nodal spread of the disease was observed. Distant metastases (𝑀1 and 𝑀2) were found in 8 patients. Thirty-six of the patients were of stage I, whereas 27 and 8 patients were of stage II and stage III, respectively.

### Immunohistochemical expression of CD44 in TGCTs and correlation with clinicopathological variables

**Fig. 2:**
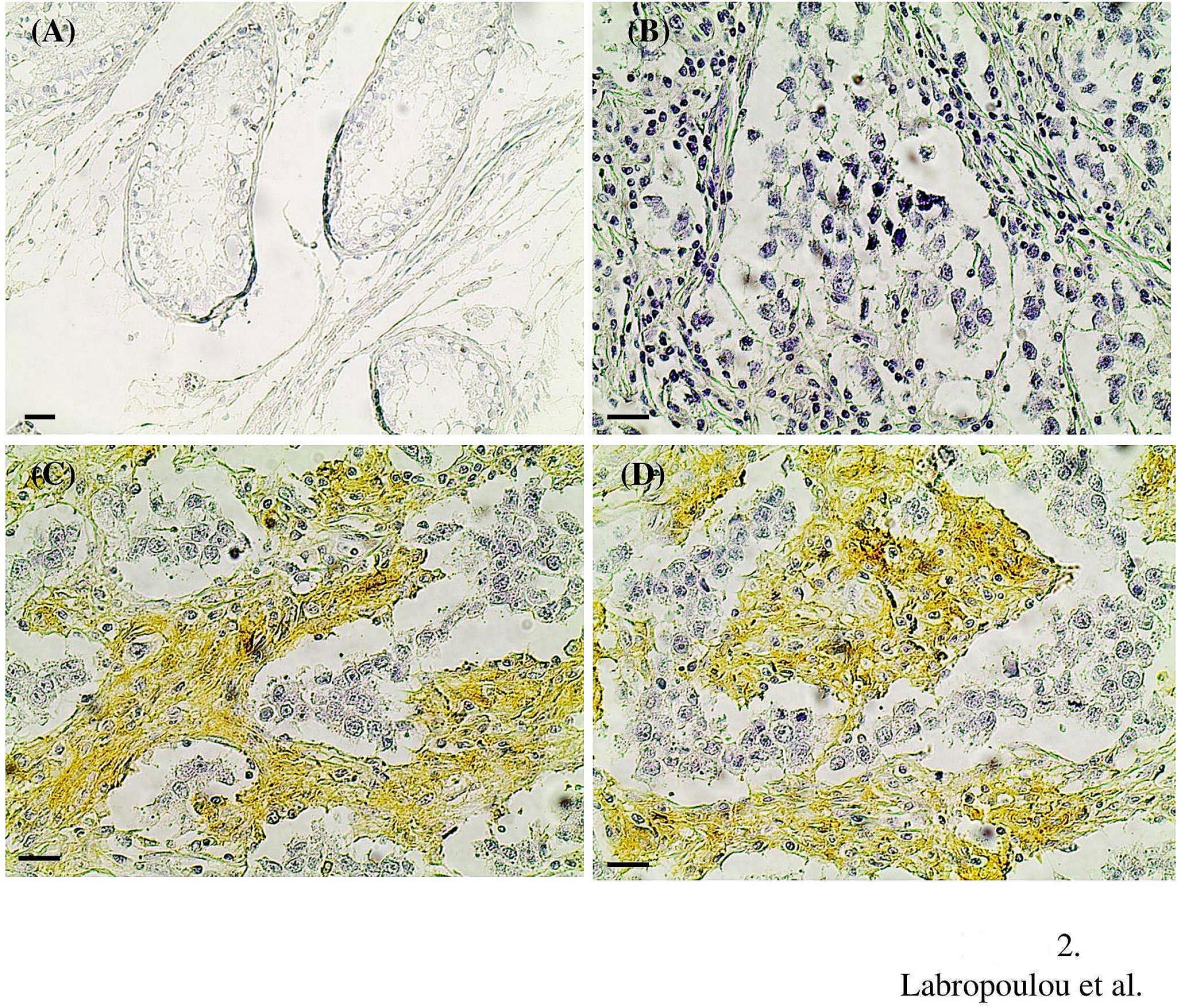
Increased expression of CD44 in advanced stage seminomas. (**A**) The seminiferous tubules and the surrounding connective tissue in the normal testicular tissue adjacent to tumor lesions were negative for the expression of CD44 as shown by immunohistochemistry. Immunohistochemical staining for CD44 in stage I (**B**) and stage II (**C** and **D**) seminomas. Positive staining for CD44 was found in tumor cells as well as in stromal components (**C** and **D**). Scale bars, 25 μm

**Fig. 3:**
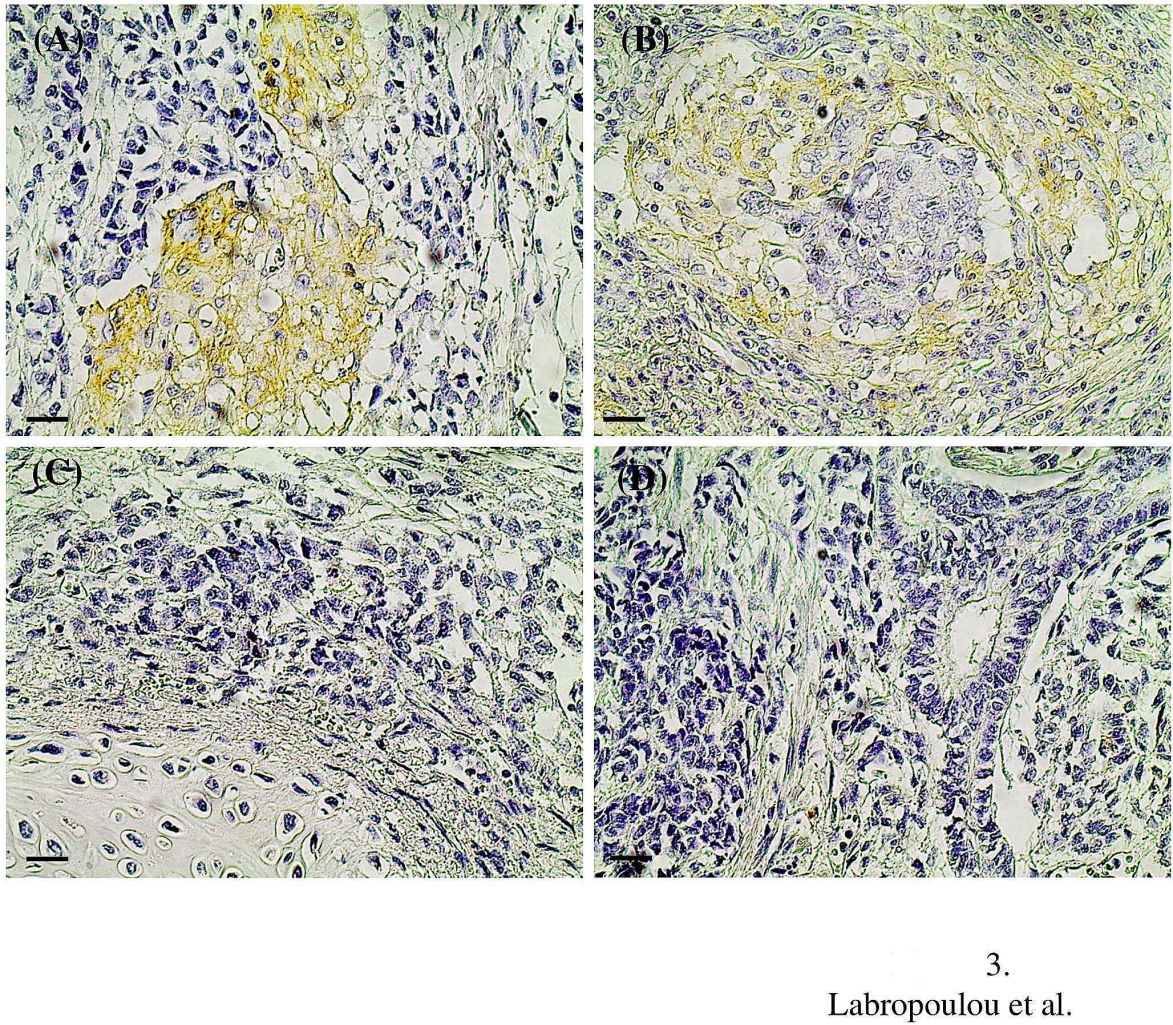
Immunohistochemical staining of CD44 in NSGCTs. (**A**) Staining for CD44 in embryonal carcinoma stage II and (**B**) in mixed type NSGCTs (embryonal and yolk sac) stage II. Absence for staining for CD44 in teratomas of stage I (**C**) and stage II (**D**). Scale bars, 25 μm

We performed immunohistochemistry in tissue sections to evaluate the expression of CD44 in tumor and stromal cells. Absence of staining for CD44 was observed in the normal seminiferous tubules adjacent to tumor lesions as well as in the interstitial connective tissue in the interlobular septa around the normal seminiferous tubules (Fig. 2). Positive staining for CD44 was found in tumor cells, stromal components, or both in seminoma (Fig. 2) and NSGCTs (Fig. 3). Most seminomas (24 out of 33) found negative for CD44 staining in tumor cells (≤10% tumor cell positivity), whereas only 9 out of 33 seminomas were positive for CD44 expression (>10% tumor cell positivity) (Table 2). Similarly, the majority of NSGCTs (24 out of 38) found negative for CD44 staining in tumor cells (≤10% tumor cell positivity), whereas 14 out of 38 NSGCTs were positive for CD44 expression (>10% tumor cell positivity) (Table 2). Positive CD44 staining was found in stromal components in 16 out of 33 patients with seminoma and in 10 out of 38 patients with NSGCTs (Table 2). CD44 staining was detected both at the cell membrane and in the cytoplasm of tumor cells in seminomas (Fig. 2) and NSGCTs (Fig. 3). According to stromal CD44 staining, both stromal cells and ECM in seminomas (Fig. 2) and in NSGCTs (Fig. 3) were positive. Positive CD44 staining in tumor cells and stromal components was associated with increased tumor size, the presence of nodal infiltration, elevated vascular/lymphatic invasion, and advanced stage of disease in seminomas (Fig. 2; Table 3). These data suggest a tumor promoting role for CD44 in tumor and stromal cells. In contrast, the elevated expression of CD44 in tumor cells and stromal components was not associated with any clinicopathological variable in NSGCTs (Fig. 3; Table 4).

**Table 2:** CD44 expression in 71 patients with testicular tumors

Histological type	CD44 positive tumor cells				CD44 stromal staining	
	<10%	10–30%	>30%		Negative	Positive
**Seminoma**	24	7	2		17	16
**NSGCTs**	24	13	1		28	10

**Table 3:** The association between CD44 stromal and tumor cells’ staining and the clinicopathologic variables of 31 patients with seminoma

(Variable)	CD44 stromal staining		Statistics		CD44 positive tumor cells		Statistics
	Negative	Positive			≤10%	>10%	
Tumour size (T)							
*T*_*1*_	11	4			14	1	
*T*_*2*_ *+ T*_*3*_	6	12	*P* = 0.04		10	8	*P* = 0.02
Nodal status (N)							
*N*_*0*_	14	6			18	2	
*N*_*1*_ *+ N*_*2*_	3	10	*P* = 0.01		6	7	*P* = 0.01
Vascular-lymphatic invasion							
*Negative*	14	7			18	3	
*Positive*	3	9	*P* = 0.03		6	6	*P* = 0.04
Disease stage							
*I*	14	6			18	2	
*II*	3	10	*P* = 0.01		6	7	*P* = 0.01

**Table 4:** The association between CD44 stromal and tumor cells’ staining and the clinicopathologic variables of 38 patients with NSGCTs

(Variable)	CD44 stromal staining		Statistics		CD44 positive tumor cells		Statistics
	Negative	Positive			≤10%	>10%	
Tumour size (T)							
*T*_*1*_	7	3			9	1	
*T*_*2*_ *+ T*_*3*_	21	7	*P* = 1.0		15	13	*P* = 0.06
Nodal status (N)							
*N*_*0*_	13	3			10	6	
*N*_*1*_ *+ N*_*2*_ *+ N*_*3*_	15	7	*P* = 0.46		14	8	*P* = 1.0
Distant metastases (M)							
*M*_*0*_	24	6			20	10	
*M*_*1*_ *+ M*_*2*_	4	4	*P* = 0.17		4	4	*P* = 0.43
Vascular-lymphatic invasion							
*Negative*	8	3			8	3	
*Positive*	20	7	*P* = 1.0		16	11	*P* = 0.49
Disease stage							
*I*	13	3			10	6	
*II + III*	15	7	*P* = 0.46		14	8	*P* = 1.0

### Stromal expression of CD44 enhances neovascularization

**Fig. 4:**
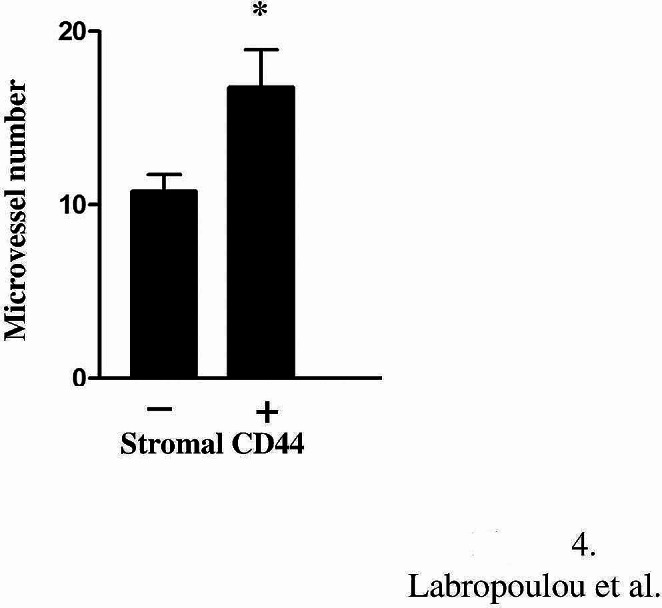
Increased expression of CD44 in tumor stroma is related to angiogenesis. The elevated number of microvessels in TGCTs is associated with CD44 staining in tumor stromal components. Asterisk (*) indicates statistically significant differences (*p* ≤ 0.05) (Student’s *t*-test)

We also examined the correlation of CD44 expression with neovascularization in tumor stroma. The increased immunoreactivity of CD44 in stromal cells and ECM components was associated with elevated microvessel number in TGCTs suggesting the involvement of CD44 in angiogenesis in disease (Fig. 4).

## Discussion

CD44 is considered as an important cell surface receptor, which is involved in cell matrix interactions and tumorigenic signaling, and regulates tumor cell phenotype, stemness and aggressiveness [12, 24]. The distinct biological features of seminomas and NSGCTs regarding the role of ECM molecules in disease progression have also emerged in previous studies [18, 19]. For example, the accumulation of versican in NSGCTs exhibits a stronger correlation with disease aggressiveness compared to seminomas [18]. Additionally, the loss of syndecan-4 expression in tumor cells in NSGCTs and the higher expression of syndecan-4 in stromal cells in seminomas are associated with metastatic potential in TGCTs [19]. Although previous studies have shown the expression of CD44 only in TGCTs and not in normal tissue, they did not investigate the association of CD44 with the features of TGCTs [25–27]. The aim of our study was to examine the expression of CD44 in different subtypes of TGCTs and tumor cell lines and to investigate the possible correlation of CD44 expression with the malignant behavior of the tumors. We found that seminomas and NSGCTs cell lines express high levels of the standards isoform of CD44. Overexpression of CD44 is also evident in analyzed tumor samples from patients. Analyses of publicly available datasets reveal elevated mRNA levels of CD44 either in seminomas or in NSGCTs compared to normal tissues. Immunohistochemical staining in patients’ material confirmed the presence of CD44 only in TGCTs and not in normal tissue. The elevated expression of CD44 in tumor and stromal cells and ECM components in seminoma is associated with tumor size, nodal metastasis, vascular/lymphatic invasion, and disease stage. In contrast, the higher expression of CD44 in NSGCTs is not correlated with the aggressive behavior of tumors in this histological subtype.

CD44 is frequently expressed in high levels in tumor cells and is considered as a cancer stem cell marker. CD44 interacts with numerous ECM components including hyaluronan, fibronectin, laminin, proteoglycans such as serglycin and biglycan, growth factors, cytokines and growth factor receptors activating oncogenic signaling that support cancer cell progression and metastasis by regulating cancer cell stemness, survival, proliferation, adhesion and invasion [12, 23, 24, 31]. Hyaluronan, which is frequently up-regulated in tumors, binds to CD44 activating various signaling cascades including MAPK, PI3K/Akt, Wnt/β-catenin, c-Src and nF-κB to promote cell survival, proliferation, and motility [32]. The levels of hyaluronan and its molecular size are regulated by the expression and the action of hyaluronan synthesizing enzymes such as hyaluronan synthases 1–3 and hyaluronan degrading enzymes including hyaluronidases. The coordinated functions of these enzymes provide varying levels and sizes of hyaluronan in the tumor microenvironment that in turn affect its interaction with CD44 and the degree and outcome of CD44 signaling in tumor and stromal cells [32]. Similarly, the abundance of other CD44 ligands in the tumor microenvironment including matrix components, growth factors and cytokines as well as the presence of cell surface receptors, which are clustered with CD44 and activate signaling pathways, are also crucial players for the activation of CD44. Although CD44 is expressed almost equally in seminomas and NSGCTs, it is correlated with aggressiveness only in seminomas. This may be attributed to the altered expression of other biological factors associated with CD44 activation and signaling in these two tumor subtypes. These subtypes, most likely, present different expression profiles of molecules involved in CD44 functional network that reflect their distinct biological features.

Receptor for hyaluronan mediated motility (RHAMM)/CD168 is another hyaluronan receptor, which is unconventionally exported and associates with the cell surface through interactions with integral cell surface proteins, including CD44, and thus functions as a co-receptor regulating cell signaling [33]. RHAMM is also a mitotic spindle-associated protein [34–36], which is highly expressed in testicular germ cells and among others controls the balance between self-renewal and differentiation of spermatogonia [37]. RHAMM expression is decreased in 90% of human seminomas and is implicated in the progression of germ cell neoplasia in situ (GCNIS) [37]. Deletion of C-terminus of RHAMM in a mouse model abolishes RHAMM association with the mitotic spindle and results in the disruption of the oriented division of undifferentiated spermatogonia and premature displacement from the basal compartment accompanied by the formation of precancerous lesions and cell atypia that could progress to seminoma [37]. It has been shown that CD44 is not expressed in normal testes but only in a subset of GCNIS testes and its genetic deletion in the RHAMM truncation background markedly reduces the percentage of mice presenting with germ cell atypia. It is suggested that overexpression of CD44 combined with RHAMM dysfunction drives oncogenesis in the testis [38].

Transformation of mouse testis cell in vitro either by lentivirus-mediated transfection of dominant negative *Trp53*, *Myc*, and *Hras1* or by transfection of *Pou5f1*, *Myc*, Klf4 and *Sox2* into CD90-expressing testis cells caused tumorigenic conversion in vitro and formation of mixed TGCTs in vivo after transplantation. The transformation of normal testis cells is accompanied by induction of CD44, which is not expressed in normal cells [39]. Overexpression of CD44 and metabolism-related proteins associated with a hyper-glycolytic phenotype and aerobic glycolysis such as glucose transporter 1 (GLUT1), monocarboxylate transporter 1 (MCT1) and monocarboxylate transporter 4 (MCT4) has been shown in TGCTs [40, 41]. Up-regulation of MCT1, MCT4 and EMMPRIN/CD147 in TGCTs is associated with aggressive clinicopathological characteristics [41]. CD44 and EMMPRIN/CD147 co-operate to transport MCT1 and MCT4 at the plasma membrane in breast cancer cells [42]. We have previously shown that EMMPRIN/CD147 is highly expressed in TGCTs cells and regulates malignant properties of the cells by regulating membrane vesicles cargo and MMP-2 secretion by stromal fibroblasts [22]. It is well known that glycolysis and lactate secretion contribute to tumor cell aggressiveness and this has been also shown in TGCTs [40]. It is likely that an additional mechanism by which CD44 together with EMMPRIN/CD147 may contribute to TGCTs aggressiveness is to regulate aerobic glycolysis in these tumors.

CD44 has also been found to be expressed by several cells in the tumor stroma including cancer-associated fibroblasts (CAFs) and endothelial cells [12, 31]. This coincides with our data on the increased expression of CD44 in tumor stromal cells and stromal components in TGCTs and the association of CD44 expression with the aggressive behavior of seminomas. CD44 is highly expressed in CAFs in hypoxic avascular but also in vascularized areas of the tumors and CD44-positive CAFs seem to connect with CD31-positive endothelial cells [43]. CD44-positive CAFs sustain the stemness and drug resistance of cancer cells [43]. Similarly, the expression of CD44 in fibroblasts supports the survival and resistance of breast cancer cells to paclitaxel through IGF2BP3-CD44-IGF2 signaling axis [44].

Additionally, a positive correlation between CD44 expression and microvessel density has been shown in numerous cancer types [31]. CD44 co-operates with multiple signaling pathways and regulates endothelial cell proliferation, adhesion and migration promoting angiogenesis [31]. For example, the extracellular part of CD44v6 expressed in endothelial cells is required for interaction with c-Met or VEGFR-2. The short endoplasmic part of CD44v6 that links ezrin and the cytoskeleton is also required for the proper signaling transmission by activated MET and VEGFR-2. CD44v6 emerges as a crucial co-receptor for c-Met and VEGFR-2 that controls endothelial cell migration, sprouting, and tubule formation in vitro and blood vessels development in vivo induced by hepatocyte growth factor (HGF) or vascular endothelial growth factor-A (VEGF-A) [45]. In agreement with the literature data, we found a positive association between increased CD44 expression in tumor stroma with a higher number of microvessels, suggesting a role for CD44 in neoangiogenesis.

## Conclusions

We demonstrate that CD44 is over-expressed in TGCTs and in tumor stroma compared to normal tissue and it is associated with diverse biological outcomes in seminomas and NSGCTs. The increased expression of CD44 in seminoma cells as well as in tumor stroma is positively associated with clinicopathological variables such as tumor size, nodal metastasis, vascular/lymphatic invasion, and disease stage. In contrast, the elevated levels of CD44 in tumor cells and in stroma in NSGCTs are not related to any clinicopathological variable. The presence of CD44 in tumor stroma of TGCTs is also correlated with neovascularization in tumor microenvironment. Our data suggest a distinct tumor-promoting role for CD44 in seminomas.

## Electronic supplementary material

Below is the link to the electronic supplementary material.

Supplementary Material 1

## Data Availability

The data presented in this study are available in this article.

## Abbreviations

ECM: extracellular matrix
EGFR: epidermal growth factor receptor
HGF: hepatocyte growth factor
HGFR/MET: hepatocyte growth factor receptor
GCNIS: germ cell neoplasia in situ
GLUT1: glucose transporter 1
GPCRs: G protein coupled receptors
MCT1: monocarboxylate transporter 1
MCT4: monocarboxylate transporter 4
MMP-2: matrix metalloproteinase-2
MMP-9: matrix metalloproteinase-9
NSGCTs: non-seminomatous germ cell tumors
PARP: poly-ADP ribose polymerase RHAMM, receptor for hyaluronan mediated motility
TGCTs: testicular germ cell tumors TLRs, toll-like receptors
VEGF-A: vascular endothelial growth factor-A
VEGFR-2: vascular endothelial growth factor receptor-2
