# Supplementary material for: Expression of CD44 is associated with aggressiveness in seminomas
Source: Mol Biol Rep. 2024 May 25;51(1):693. doi: 10.1007/s11033-024-09638-8 (PMC11127849; doi:10.1007/s11033-024-09638-8)
Supplement: Supplementary file 1 — Supplementary Material 1 [file 11033_2024_9638_MOESM1_ESM.docx]

**Expression of CD44 is associated with aggressiveness in seminomas**

Vasiliki T. Labropoulou^1*^, Dimitra Manou^2^, Panagiota Ravazoula^3^, Fatimah Mohammed Alzahrani^4^, Haralabos P. Kalofonos^5^, Achilleas D. Theocharis^2,4*^

^1^Department of Internal Medicine, Division of Hematology, University of Patras Medical School, Patras, Greece

^2^Biochemistry, Biochemical Analysis and Matrix Pathobiology Research Group, Laboratory of Biochemistry, Department of Chemistry, University of Patras, Greece

^3^Department of Pathology, University Hospital of Patras, 26504 Patras, Greece

^4^Department of Chemistry, College of Science, Princess Nourah bint Abdulrahman University, P.O. Box 84428, Riyadh 11671, Saudi Arabia

^5^Clinical Oncology Laboratory, Division of Oncology, Department of Medicine, University of Patras, Rio, 26504, Greece

**Supplementary Figure S1A.** CD44 expression in 68 seminomas and 68 NSGCTs according to GEPIA2 (Gene Expression Profiling Interactive Analysis 2, <http://gepia2.cancer-pku.cn/#index>). No significant differences in the expression of CD44 are present between seminomas and NSGCTs

**Supplementary Figure S1B.** Expression-methylation correlation analyses for CD44 in 63 seminomas was obtained by cbioportal (https://www.cbioportal.org/, Human Methylation 27 (HM27) and Human Methylation 450 (HM450) merged arrays, Testicular Germ Cell Tumors, TCGA, PanCancer Atlas) [28]. Methylation levels are reported as beta-values and gene expression as mRNA expression z-scores relative to all samples (log RNA Seq V2 RSEM). The line was modeled through simple linear regression and the statistic tests were Spearman and Pearson correlation tests.

**Supplementary Figure S1C.** Expression-methylation correlation analyses for CD44 in 86 NSGCTs was obtained by cbioportal (https://www.cbioportal.org/, Human Methylation 27 (HM27) and Human Methylation 450 (HM450) merged arrays, Testicular Germ Cell Tumors, TCGA, PanCancer Atlas) [28]. Methylation levels are reported as beta-values and gene expression as mRNA expression z-scores relative to all samples (log RNA Seq V2 RSEM). The line was modeled through simple linear regression and the statistic tests were Spearman and Pearson correlation tests.

**Supplementary Figure S1D.** Western blot and densitometric analysis of protein levels of CD44 in JKT-1 seminoma, NCCIT and NTERA-2/D1(NT2/D1), embryonal carcinoma cells.
